# Religion, a social determinant of mortality? A 10-year follow-up of the Health and Retirement Study

**DOI:** 10.1371/journal.pone.0189134

**Published:** 2017-12-20

**Authors:** Ellen Idler, John Blevins, Mimi Kiser, Carol Hogue

**Affiliations:** 1 Department of Sociology, Emory University, Atlanta, Georgia, United States of America; 2 Hubert Department of Global Health, Rollins School of Public Health, Emory University, Atlanta, Georgia, United States of America; 3 Department of Epidemiology, Rollins School Public Health, Emory University, Atlanta, Georgia, United States of America; Tulane University School of Public Health and Tropical Medicine, UNITED STATES

## Abstract

The social determinants of health framework has brought a recognition of the primary importance of social forces in determining population health. Research using this framework to understand the health and mortality impact of social, economic, and political conditions, however, has rarely included religious institutions and ties. We investigate a well-measured set of social and economic determinants along with several measures of religious participation as predictors of adult mortality. Respondents (N = 18,370) aged 50 and older to the Health and Retirement Study were interviewed in 2004 and followed for all-cause mortality to 2014. Exposure variables were religious attendance, importance, and affiliation. Other social determinants of health included gender, race/ethnicity, education, household income, and net worth measured at baseline. Confounders included physical and mental health. Health behaviors and social ties were included as potential explanatory variables. Cox proportional hazards regressions were adjusted for complex sample design. After adjustment for confounders, attendance at religious services had a dose-response relationship with mortality, such that respondents who attended frequently had a 40% lower hazard of mortality (HR = 0.60, 95% CI 0.53–0.68) compared with those who never attended. Those for whom religion was “very important” had a 4% higher hazard (HR = 1.04, 95% CI 1.01–1.07); religious affiliation was not associated with risk of mortality. Higher income and net worth were associated with a reduced hazard of mortality as were female gender, Latino ethnicity, and native birth. Religious participation is multi-faceted and shows both lower and higher hazards of mortality in an adult US sample in the context of a comprehensive set of other social and economic determinants of health.

## Introduction

The social determinants of health framework is the current dominant paradigm in public health and epidemiology. It originated in the 1960s with the Whitehall Studies of British civil servants, which established that there was a social gradient of health inequality stretching across all social strata within this single work sector [1]. Further studies of data from England and Wales and the United States confirmed health inequality gradients tied to socioeconomic indicators of income and education in large, representative populations [2]. Archival data from longitudinal studies beginning in childhood underscored the importance of early life experiences and their influence on later life health [3]. In 2008 the World Health Organization’s Commission on Social Determinants of Health published a report arguing that worldwide health inequalities arise from “…the circumstances in which people grow, live, work, and age….” The Commission further argued that “…the conditions in which people live and die are, in turn, shaped by political, social, and economic forces.” Thus there has been a pronounced recent public health emphasis on the upstream social conditions that are "fundamental causes" [4] of health throughout the life course.

Simultaneously, religious involvement as a factor in health has been studied for decades [5, 6]. Systematic reviews [7, 8] and meta-analyses [9] have concluded that attendance at religious services has a robust association with reduced mortality in adult populations, and to a lesser extent outcomes such as functional disability. Although religious participation levels vary from one society to another, religious institutions are present to some extent in all societies, and they are particularly prominent in the global south [10, 11]. Thus it is somewhat surprising that those who study the social sources of health inequalities, especially those with a focus on global health, have almost completely omitted a discussion of religion as one of the social determinants of health.

Religious participation in the social form of attendance at religious services may have a positive impact on individual health outcomes through its provision of social ties and social support, its influence on health risk and health promoting behaviors, and/or the access it provides to the social capital of religious groups [12]. Other more private or subjective dimensions of religious involvement such as prayer, religious beliefs, religious coping, or the importance of religion have shown less consistent associations with better health outcomes, and in some cases are associated with poorer health [8]. There are relatively few studies of religion as a predictor of mortality that include measures of multiple dimensions of religion [13].

To consider religion among the social determinants of health, stronger measures of the socioeconomic determinants of health inequalities are also required. While studies of religion as a predictor of mortality have usually included covariates for education and sometimes included measures of income, none have included measures of household wealth, which is seldom available in epidemiologic studies [14], given the difficulty of collecting such complex data. Accumulated wealth and assets are far greater sources of economic inequality than annual income, which is even less an indicator of economic status for older populations in retirement [15, 16, 17]. To our knowledge, no nationally representative analyses of US adult mortality have included multiple measures of religion and detailed measures of socioeconomic status, including both income and wealth. Such an analysis would contribute to understanding religion as a social determinant of health in conjunction with those social determinants that have defined the framework to date. Our objective in this paper is to test the association of both private and public forms of religious participation with survival, in the context of a comprehensive set of social and economic determinants of health.

## Methods

### Study population

Data are drawn from the Health and Retirement Study (HRS), a biennial survey of adults aged 41 and older that is nationally representative of non-institutionalized US adults. The HRS began in 1992 as a study of health and economic circumstances associated with aging among adults aged 51–61, and since then has expanded to include both older and younger adults. Details of the multistage sample design are available elsewhere [18, 19]. African-American and Hispanic households are oversampled at about twice the rate of white households; most interviews are conducted face-to-face. The survey includes weights for sample design and nonresponse. We used 2004 as our baseline because multiple questions about religious involvement were introduced in that year and the sample first included early baby boomers (birth cohorts 1948–53). The 2004 sample size is 20,192, but dropping persons with invalid weights due to out of range birth years or nursing home residence, yields a maximum analytic sample size incorporating weights of 18,701.

### Measurements

#### Mortality

The outcome variable was mortality from all causes. Mortality follow-up to 2014 consists of National Death Index (NDI) records for month and year of death through 2011, linked to HRS records through identifying variables and released with the public use data set. We supplemented NDI records with household contact reports of death obtained at biennial follow-ups; these were almost exclusively deaths that took place subsequent to the most recent NDI records (2012–14).

#### Religion

Three measures of religious involvement were our primary independent variables. Respondents were asked their religious affiliation, which HRS grouped as Mainline Protestants, Conservative Protestants, Roman Catholics, Jews, other religion, and no religion. HRS coded "How often have you attended religious services in the past year" in five categories from never to more than once per week; we reduced these categories to four, for "frequently" (more than once per week), "regularly" (2–4 times per month), "occasionally" (1–12 times per year) and "never". We retained the HRS coding of "How important is religion in your life?" in three categories from "not too important" to "very important".

#### Demographics

Demographic characteristics include age (continuous); female gender; self-reported race (white, black, other); self-reported Hispanic/Latino ethnicity; and US native born.

#### Socioeconomic status

Education is recorded in years (0–17). The HRS has pioneered an "unfolding bracket" method of eliciting information on income, assets, and debt that assists respondents to derive a value, for instance, for the current amount of their home loan by asking "is the amount more than, less than, or about ___", repeating the question 2–4 more times to arrive at a closely-estimated value [20]. We used these HRS "imputed" values for household income and household net worth (sum of all assets and debts), dividing them into quartiles.

#### Health status

Health status measures included a count of chronic conditions that can be causes of death (diabetes, cancer, lung disease, heart disease, stroke) and a count of non-cause-of-death conditions (high blood pressure, arthritis, other conditions). Other measures included days in bed in the last month, self-rated health, a count of symptoms (e. g. incontinence, swelling, shortness of breath), pain, a count of Activities of Daily Living (ADLs, 0–9), a count of Instrumental Activities of Daily Living (IADLs, 0–14), and sensory (vision and/or hearing) impairment. Measures of mental health included the Center for Epidemiologic Studies’ Depression scale (9 items scored 0–1, mean of nonmissing items if < 4 missing), self-reported emotional or psychiatric problems, and self-reported memory problems.

#### Health behaviors

Health behaviors included smoking (ever, never, current), alcohol use (usual days per week), body mass index (weight/height^2^ categorized as underweight, normal weight, overweight, obese I, obese II), physical exercise (vigorous, moderate, mild), and a count of health promotion/prevention activities (annual flu shot, cholesterol screen, mammogram, prostate exam, seat belt use).

#### Social ties

Social ties included marital status (married, widowed, divorced/separated, never married), family size (sum of children, grandchildren, siblings, parents), frequency of socializing, and volunteering/caregiving.

### Data analysis

We used Stata Version 14.2 for all analyses. All analyses were weighted and had standard errors adjusted for the complex sample design. We used Cox proportional hazards models to predict mortality as of 2014 according to all three religion measures (affiliation, attendance, importance), then introduced other demographic and economic social determinants of health, health status confounders, and health behaviors and social ties as potential explanatory variables. Table 1 shows our analytic strategy for adding blocks of variables in steps. Table 2 summarizes data for all measures at 2004 baseline. Table 3 shows models 1–3 for religion and the other social determinants. Table 4 shows results for only the religion measures in models 1–6 and model fit statistics. Bivariate associations of all variables with religious attendance are shown in S1 Table and with importance of religion in S2 Table. Complete versions of models 1–6 are shown in S3 Table. Gender-specific models are shown in S4 Table.

**Table 1:** Modeling steps of the Cox proportional hazard regressions, Health and Retirement Study 2004–2014.

Model	Description	Variables
1	Baseline	Religious participation, religious importance, religious affiliation
2	Demographics	Baseline + Age, gender, race, Latino, US born
3	SES	Baseline + Demographics + Education, household income, household net worth
4	Health status	Baseline + Demographics + SES + Chronic conditions (cause of death count, non-cause of death count), bed days, self-rated health, symptoms, pain, ADL, IADL, sensory impairment, CESD, emotional problems, memory problems
5	Health behaviors	Baseline + Demographics + SES + Health status + BMI (underweight, overweight, obese I, obese II), smoker (past, current, never), alcohol (days per week), exercise, health promotion/prevention count
6	Social ties	Baseline + Demographics + SES + Health status + Marital status, family size, socialize frequently, volunteering

Note: All models are survey-design adjusted, including weighting and adjusted standard errors.

**Table 2:** Descriptive statistics, Health and Retirement Study 2004, weighted and adjusted for complex sample design.

	Full Sample	Male	Female
Variable	% / mean (se)	% / mean(se)	% / mean (se)
**Religion**			
Attends frequently	13.90	11.50	15.92
Attends regularly	36.78	33.19	39.82
Attends occasionally	23.00	24.53	21.65
Never attends	26.35	30.78	22.61
Religion is "very important"	64.79	54.85	73.18
Mainline Protestant	25.35	21.28	28.78
Conservative Protestant	36.27	36.09	36.42
Roman Catholic	27.63	27.68	27.58
Jewish	2.45	2.43	2.46
Other religion	1.51	1.64	1.41
No religion	9.24	12.34	6.63
**Demographic characteristics**			
Age in years at 2004 interview (range 50–107)	64.35 (.09)	63.51 (.12)	65.05 (.12)
Gender (female)	54.20	-	100.00
Race			
White	85.55	86.09	85.10
African-American	9.49	8.74	10.13
Other race	4.95	5.18	4.76
Latino	7.30	7.34	7.27
US Born	90.87	91.19	90.61
**Socioeconomic characteristics**			
Education in years (range 0–17)	12.74 (.02)	12.95 (.04)	12.56 (.03)
Household income in $ (range 0–3.6M)	67.14K (908.68)	76.62K (1.49K)	59.13K (1.10K)
Household assets in $ (range -499K-76.6M)	294.22K (12.73K)	337.09K (21.10K)	257.99K (15.32K)
Household net worth in $ (range -499K-77.2M)	454.34K (13.78K)	508.18K (22.72K)	408.84K (16.69K)
**Chronic conditions**			
Chronic conditions (cause of death) count (range 0–5)	0.64 (.01)	0.67 (.01)	0.61 (.01)
Chronic conditions (non-cause of death) count (range 0–3)	1.10 (.01)	1.02 (.01)	1.18 (.01)
Bed days (range 0–31)	0.70 (.03)	0.57 (.04)	0.81 (.04)
Self-rated health (range 1–5)	3.21 (.01)	3.23 (.01)	3.19 (.01)
Symptoms (range 0–7)	1.09 (.01)	0.85 (.02)	1.29 (.02)
Pain (range 0–2)	0.69 (.01)	0.64 (.01)	0.74 (.01)
**Functional limitations**			
ADL (range 0–9)	1.11 (.01)	0.91 (.02)	1.28 (.02)
IADL (range 0–14)	2.58 (.02)	2.06 (.03)	3.02 (.03)
Sensory impairment (range 0–2)	0.39 (.01)	0.50 (.01)	0.36 (.01)
**Mental health**			
CESD (range 0–1)	0.21 (.00)	0.18 (.00)	0.23 (.00)
Emotional problems	16.91	12.17	20.92
Memory problems	1.81	1.79	1.83
**Health behaviors**			
BMI (range 9.61–71.32)	27.48 (.05)	27.66 (.07)	27.33 (.07)
Underweight (BMI<18.5)	1.92	1.09	2.65
Normal weight (18.5< = BMI< = 25)	33.51	27.69	38.55
Overweight (25<BMI< = 30)	37.99	45.66	31.34
Obese I (30<BMI< = 35)	17.50	18.25	16.84
Obese II (35<BMI)	10.64	7.72	13.11
Smoking			
Never	42.66	32.42	51.31
Current smoker	16.23	18.20	14.56
Former smoker	41.10	49.34	34.13
Alcohol used days per week (range 0–7)	1.19 (.02)	1.61 (.03)	0.84 (.02)
Exercise (range 3–15)	8.55 (.02)	8.70 (.04)	8.42 (.03)
Health promotion count (range 0–4)	2.71 (.01)	2.66 (.01)	2.76 (.01)
**Social ties**			
Marital status			
Married	64.99	75.75	55.90
Never married	4.07	4.54	3.67
Widowed	16.28	6.61	24.45
Divorced/separated	14.66	13.11	15.97
Family size (range 0–99)	53.97 (.39)	67.16 (.58)	43.06 (.50)
Socialize at least weekly	52.11	54.63	49.98
Volunteer	64.40	68.75	60.72
**Mortality**			
Survived to 2014	70.60	67.74	73.35
Number of deaths	5917	2589	2845
Observations, unweighted	18,701	8025	10,676
Population size	80,221,947	36,739,650	43,482,297

Note: Frequent attendance at religious services is more than once per week; regular attendance is two times per month to weekly; occasional attendance is less than two times per month but at least once per year.

Note: Mainline Protestant includes HRS category for Reformation Era Protestants; Conservative Protestant includes HRS categories for Pietistic, Fundamentalist, General (includes Evangelical).

Note: Cause of death chronic conditions include diabetes, cancer, lung disease, heart disease, stroke; Non-cause of death chronic conditions include hypertension, arthritis, other conditions.

Note: ADL = Activities of Daily Living; IADL = Instrumental Activities of Daily Living; CESD = Centers for Epidemiologic Studies Depression scale; BMI = Body Mass Index.

Note: Health promotion activities include flu shot, cholesterol test, mammogram/prostate screening, seat belt use.

Note: Family size includes sum of children, grandchildren, brothers, sisters, mother, father.

Note: Volunteer includes ever doing informal caregiving or volunteering for organizations.

**Table 3:** Proportional hazards models for religion measures, and other social determinants of health, with adjustment for complex sample design, Health and Retirement Study, 2004–14.

	Religion	Demographics	SES
Variable	HR (95% CI)	HR (95% CI)	HR (95% CI)
**Religion**			
Participation			
Frequently	0.43*** (0.39, 0.49)	0.41*** (0.36, 0.45)	0.48*** (0.43, 0.54)
Regularly	0.50*** (0.46, 0.54)	0.51*** (0.47, 0.56)	0.60*** (0.55, 0.65)
Occasionally	0.57*** (0.52, 0.63)	0.71*** (0.65, 0.78)	0.78*** (0.71, 0.85)
Never (ref.)			
Religion is "very important"	1.13*** (1.09, 1.16)	1.10*** (1.07, 1.13)	1.06*** (1.04, 1.09)
Affiliation			
Mainline Protestant	3.93*** (3.53, 4.37)	0.99 (0.89, 1.10)	0.97 (0.87, 1.08)
Conservative Protestant	1.86*** (1.64, 2.12)	1.14* (1.02, 1.28)	1.06 (0.95, 1.18)
Roman Catholic	1.77*** (1.59, 1.96)	1.06 (0.97, 1.17)	1.03 (0.96, 1.12)
Jewish	1.65*** (1.34, 2.03)	0.84 (0.69, 1.02)	0.92 (0.75, 1.12)
Other religion	1.05 (0.74, 1.50)	0.87 (0.62, 1.23)	0.88 (0.62, 1.23)
No religion (ref.)			
**Demographic characteristics**			
Age in years		1.10*** (1.09, 1.10)	1.09*** (1.09, 1.10)
Gender (female)		0.70*** (0.66, 0.75)	0.63*** (0.59, 0.68)
Race			
White (ref.)			
African-American		1.38*** (1.26, 1.52)	1.07 (0.97, 1.19)
Other race		1.04 (0.85, 1.27)	0.92 (0.75, 1.13)
Latino		1.07 (0.92, 1.23)	0.81* (0.69, 0.95)
US Born		0.93*** (0.90, 0.96)	0.93*** (0.90, 0.96)
**Socioeconomic characteristics**			
Education in years			0.99 (0.98, 1.00)
Household income (in quartiles)			0.86*** (0.83, 0.90)
Household net worth (in quartiles)			0.84*** (0.81, 0.87)
F (df), Prob > F	136.78 (9)***	232.24 (15)***	224.53 (18)***
Observations, weighted	78,317,290	78,254,220	78,107,773
Observations, unweighted	18,321	18,298	18,274

* p < .05.

** p < .01.

*** p < .001.

**Table 4:** Mortality hazard ratios from Cox proportional hazard models for religion measures, before and after inclusion of other social determinants, potential confounders, and potential mediators, with adjustment for complex sample design, Health and Retirement Study, 2004–14.

	Religion only	+Demo-graphics	+SES	+Health status	+Health behaviors	+Social ties
Variable	HR (95% CI)	HR (95% CI)	HR (95% CI)	HR (95% CI)	HR (95% CI)	HR (95% CI)
*Religious participation*						
Frequently attend	0.43*** (0.39, 0.49)	0.41*** (0.36, 0.45)	0.48*** (0.43, 0.54)	0.60*** (0.53, 0.68)	0.74*** (0.65, 0.84)	0.65*** (0.57, 0.74)
Regularly attend	0.50*** (0.46, 0.54)	0.51*** (0.47, 0.56)	0.60*** (0.55, 0.65)	0.73*** (0.66, 0.81)	0.85** (0.77, 0.94)	0.77*** (0.69, 0.85)
Occasionally attend	0.57*** (0.52, 0.63)	0.71*** (0.65, 0.78)	0.78*** (0.71, 0.85)	0.85** (0.77, 0.95)	0.92 (0.83, 1.02)	0.87* (0.78, 0.97)
Never attend (ref.)						
*Importance of religion*						
Religion is "very important"	1.13*** (1.09, 1.16)	1.10*** (1.07, 1.13)	1.06*** (1.04, 1.09)	1.04*** (1.01, 1.07)	1.04* (1.01, 1.07)	1.04* (1.00, 1.07)
*Affiliation*						
Mainline Protestant	3.93*** (3.53, 4.37)	0.99 (0.89, 1.10)	0.97 (0.87, 1.08)	0.96 (0.85, 1.08)	0.92 (0.81, 1.04)	0.94 (0.83, 1.07)
Conservative Protestant	1.86*** (1.64, 2.12)	1.14* (1.02, 1.28)	1.06 (0.95, 1.18)	1.03 (0.91, 1.16)	1.02 (0.90, 1.16)	1.05 (0.92, 1.19)
Roman Catholic	1.77*** (1.59, 1.96)	1.06 (0.97, 1.17)	1.03 (0.96, 1.12)	1.03 (0.92, 1.15)	0.99 (0.88, 1.11)	1.00 (0.89, 1.12)
Jewish	1.65*** (1.34, 2.03)	0.84 (0.69, 1.02)	0.92 (0.75, 1.12)	0.88 (0.70, 1.09)	0.90 (0.73, 1.16)	0.83 (0.67, 1.04)
Other religion	1.05 (0.74, 1.50)	0.87 (0.62, 1.23)	0.88 (0.62, 1.23)	0.74 (0.51, 1.08)	0.74 (0.50, 1.10)	0.80 (0.55, 1.17)
No religion (ref.)						
F (df), Prob > F	136.78 (9)***	232.24 (15)***	224.53 (18)***	130.14 (30)***	102.02 (39)***	106.27 (36)***
Observations, weighted	78,317,290	78,254,220	78,107,773	71,365,979	69,977,879	67,211,961
Observations, unweighted	18,321	18,298	18,274	16,595	16,269	15,698

* p < .05.

** p < .01.

*** p < .001.

## Results

Characteristics of the 2004 HRS sample are shown in Table 2. Household (spouse) respondents younger than 50 are dropped because the HRS gave them a weight of 0. The remaining data are weighted and adjusted for the complex survey design. The average age of the sample is 64.4; it is over 85% white and over 90% native born. Proportions of African-Americans, those of other race, and Latinos were oversampled, but once weighted reflect their accurate proportions for this older age sample. Respondents appear relatively well-off in terms of mean levels of income, assets, and net worth, but the actual ranges for these variables are wide and skewed; thus we created quartiles for each of these variables. Because assets and net worth were highly correlated we retained only net worth (as well as income) for the multivariate analyses. Women have nearly equivalent levels of education with men, but considerably lower income, assets, and net worth. Physical health, mental health, and disability measures for this older but non-institutionalized population are also good, with men generally reporting somewhat better health and fewer functional limitations than women.

Proportions of HRS respondents identifying as Protestant, Catholic, Jewish, other religion, and no religion mirror those proportions for older persons in the US; fewer women than men identify as having no religion. About 14% of the sample reports attending religious services more than once per week, 37% attend at least twice per month, 23% attend occasionally, and 26% never attend; women are more likely than men to attend frequently. About 65% say that religion is “very important” to them, women much more likely than men.

There were 5917 deaths between 2004 and 2014. 70.6% of the sample survived, 73.3% of women and 67.7% of men.

Health behaviors are a potential explanatory pathway for the association between the measures of religion and mortality if those who are more religiously observant also observe better health behaviors. Nearly two-thirds of the sample is overweight or obese, men moreso than women. Over half are current or former smokers, and again men have higher rates. On the other hand, alcohol use is low, exercise is in the moderate range, and respondents reported on average 2.7 out of a possible 4 health promotion behaviors.

Social ties are another potential explanatory pathway, if religious participation is associated with a greater number of social ties. Over half of the sample are married, socialize at least once per week, and volunteer or provide caregiving. Men are more likely to be married and women more likely to be widowed. Men report larger family size, and more socializing and volunteering.

To better understand the cross-sectional correlates of 2004 religious attendance, we ran ordinary least squares regressions for blocks of variables, with attendance as the dependent variable (S1 Table). Frequency of attendance is positively associated with importance of religion, and is higher for Conservative Protestants and Catholics, compared with Mainline Protestants, but lower for Jews and those with no religion. Older persons, women, African Americans, and Latinos show significantly higher levels of attendance. Attendance is associated positively with years of education, household income in quartiles, and household net worth. The association of attendance with health is mixed; respondents with higher levels of attendance reported more chronic conditions and IADL difficulties, but also better self-rated health, less pain, fewer ADL difficulties, less sensory impairment, and fewer depression symptoms and emotional problems. Those who attend more often are significantly less likely to be current smokers and to drink frequently, and are more likely to exercise and do health promotion activities. There is also a mixed picture with respect to social ties. Married persons attend less frequently than widowed persons, but more often than the divorced and never married. Attendance is higher for those with smaller family sizes, those who socialize frequently and those who volunteer. Overall, cross-sectionally, those who attend religious services more often show both advantages and disadvantages with respect to other social determinants of health, indicators of health status, health behaviors, and social ties.

We performed the same analyses for the subjective perception of religious importance (S2 Table). Again, importance of religion is associated with a higher level of attendance, and Conservative Protestants find more importance in religion than Mainline Protestants do, but Jews and those with no religion find less; Roman Catholics and those with other religions are similar to Mainline Protestants. Older persons, females, African-Americans and those of other races (compared to whites), and Latinos report greater importance of religion, and US born respondents report less than the non-native born. Respondents with higher levels of education and net worth report less importance. The picture is mixed with respect to health status; greater importance of religion is associated with higher levels of non-cause-of-death chronic conditions, symptoms, and IADLs, but with fewer reports of pain, and it is not associated at all with the other health status measures. Current smokers and those with more health promotion activities find greater importance in religion, and those with higher alcohol use and less exercise find it less important. Finally, importance of religion is associated positively with being widowed, divorced/separated, socializing frequently, and volunteering, and associated negatively with larger family size. In sum, religious attendance and the importance of religion to the respondent—while positively associated with each other—show somewhat different associations with other social determinants of health and especially with health status. Cross-sectionally, higher levels of religiousness are by no means always associated with better health.

Table 3 shows the results of Cox proportional hazards models for the three religion measures and subsequent additions of the other social determinants of demographic characteristics and socioeconomic status. With only the religion variables in the model, more than weekly attendance at services shows a 57% lower hazard of mortality compared with those who never attend (HR = 0.43, 95% CI = 0.39, 0.49). Other categories of attendance also showed significantly reduced hazards of 50% and 47%. Respondents who said that religion was “very important”, however, had a 13% higher hazard (HR = 1.13, 95% CI = 1.09, 1.16). And compared with no religious affiliation, all affiliations except “other religion” had significantly higher hazards. Adding demographic characteristics shows that older persons, men, African-Americans, and the non-native-born have higher hazards of mortality. The addition of these social determinant variables increases the protective effect for those who attend more than weekly to a 59% lower hazard (HR = 0.41, 95% CI = 0.36, 0.45) and eliminates most differences for religious affiliations. Additional analyses that looked separately at the individual demographic variables show that the age of the respondent is entirely responsible for the nearly complete mediation of the religious affiliation variables; only Conservative Protestants remain with a significantly higher hazard.

When socioeconomic characteristics are added, higher household income (HR = 0.86, 95% CI = 0.83, 0.90) and net worth (HR = 0.84, 95% CI = 0.81, 0.87), but not years of education are significantly associated with survival; this additional set of social determinants slightly reduces the protective association for frequent attendance to a 52% lower hazard (HR = 0.48, 95% CI = 0.43, 0.54) and slightly diminishes the still-higher hazard ratio for importance of religion (HR = 1.04, 95% CI = 1.04, 1.09). It also completely mediates the remaining significant association for Conservative Protestants; a closer look shows that both household income and net worth mediate the effect on their own. In supplemental bivariate analyses, Conservative Protestants were considerably less likely than any other religious affiliation to be in the top quartile for household net worth (just 19%), and were much more likely than the other groups (41.2%) to be in the bottom quartile. In this case, then, mortality hazard differences by religious affiliation appear to be explained by the lower socioeconomic status of Conservative Protestants.

Table 4 continues the modeling strategy laid out in Table 1, but showing only the hazard ratios for the three religion measures (complete models are shown in S3 Table). Health status measures are added in the next step. They are potential confounders for attendance in that respondents who are ill might be less able to travel to religious services. Higher numbers of cause-of-death chronic conditions, bed days, and IADLs, and poorer self-rated health are all associated with higher hazards of mortality; however, non-cause-of-death chronic conditions, pain, and sensory impairment show significantly lower hazards. Together these health status measures reduce the protective association for frequent attendance to a 40% lower hazard (HR = 0.60, 95% CI = 0.53, 0.68) but all levels of attendance retain significantly lower hazards in a dose-response fashion.

In the next step we added health behaviors to see if the better health behaviors of those who attend services more often would explain some of the association (complete model shown in S3 Table). Current and former smoking, and being underweight all have higher hazards, while overweight, exercise, and health promotion behaviors all have lower hazards. The addition of this set reduces the protective association for frequent attendance to 0.74 (95% CI = 0.65, 0.84) and reduces the protective association of occasional attendance to nonsignificance. Additional analyses showed that the mediated effect is due mostly to smoking (attenders smoke less) and also to higher levels of exercise and prevention behaviors, and not at all to BMI or alcohol use.

Alternatively, we added the potential mediator of social ties to the model (complete model shown in S3 Table). Only volunteering was significant, reducing the hazard of mortality by 21% compared with those who did not volunteer. This set of variables reduced the protective association for frequent attendance to 0.65 (95% CI = 0.57, 0.74), but all levels remain significant.

In both of these final models, respondents who reported that religion was very important to them had a statistically significant HR of 1.04, meaning a 4% higher hazard of mortality. Additional analyses were conducted to understand this finding. Respondents who said religion was very important to them but who never attended services were older, more likely to be female, more likely to be widowed, had higher ADL counts, higher CESD scores, and were more likely to have died by 2014 than the sample as a whole. In a model estimating specific contrasts, the never attend/very important group had a significantly higher hazard of mortality than the comparison group of never attend/not at all important in models including demographics and SES measures (HR 1.34, 95% CI 1.16, 1.54), but this hazard was no longer significant when health status measures were included in the model (HR 1.16, 95% CI .99, 1.37), suggesting a heightened importance of religion for those in poor health and nearer the end of life.

### Sensitivity analyses

To address possible gender differences in the relationship between religion and mortality, we ran all models separately for males and females. The findings were not very different from the pooled model, although the hazards were somewhat greater for females and reduced for males. Hazard ratios across the six models for frequent attendance ranged from 0.48 to 0.81 for males, and with no confidence interval including 1.00. For females, hazard ratios for frequent attendance ranged from 0.35 to 0.70, again with no confidence interval including 1.00. A dose-response relationship was maintained in all models (complete results are shown in S4 Table). In models including an interaction term for gender*attendance in which never-attending males are the comparison group, all other groups have significantly lower (more protective) HRs in dose-response fashion.

### Causation

To rule out potential bias from unmeasured poor health status at baseline, we conducted additional analyses. Participants who were deceased in the period shortly after their baseline interview might have been ill and therefore unable to attend religious services. Conversely they may have been seeking solace from religion in a time of illness and therefore found religion to be of heightened importance. To address these possibilities we re-ran model 4 (adjusting for health status) and dropped respondents whose death occurred before January 1, 2005 (158 deaths). The hazard ratios for attendance at services and importance of religion were almost identical to those in the full sample. We also ran the model dropping respondents whose death occurred before January 1, 2006 (457 deaths) with the same result; thus we conclude that bias due to reverse causation, or unmeasured health status, is minimal or absent.

## Discussion

As have the small number of previous investigations with US nationally-representative samples, this study shows a protective effect of religious attendance against all-cause mortality [21, 22, 23]. In addition, it employs better measures of income and wealth and other social determinants of health than other studies have had available. In this context, religious attendance shows protective associations with survival time that are similar to, or larger in size and significance than these other determinants. In fully adjusted health status models, HRS respondents who attended religious services more than weekly had a 40% reduced hazard of mortality compared to those who never attended. By comparison, an additional quartile of household wealth reduced the hazard ratio 10%, an additional quartile of income by 8%, and female sex by 40%.

The protective effect of frequent attendance is partially mediated by health behaviors and to a lesser extent, social ties, a finding again similar to that of other studies [21, 22, 23, 24, 25]. Frequent attenders are less likely to be smokers or to use alcohol, and are more likely to exercise and report health promotion activities (S1 Table); together these factors account for 23% of the effect of frequent attendance on mortality (protective HR reduced from 0.60 to 0.74). Social ties accounted for 8% of the association (protective HR reduced from 0.60 to 0.65). Thus some but not all of the protective effect of frequent attendance is due to healthier lifestyles and/or greater social involvement.

Another finding very much in line with those of other studies is the *higher* hazard of mortality for those who report that religion is “very important” to them [19]. This is a frequent finding in studies—particularly in old age populations—with functional disability or morbidity outcomes [25, 26]. An increased feeling of the importance of religion in old age may coincide with illness, physical decline, and a resulting need for comfort or consolation [27]. This finding also comports well with the conclusion of several reviews of the religion and mortality literature, namely that attendance at religious services is a robust predictor of survival in studies of "healthy" populations but not in patient populations based on nonrepresentative samples [7, 8, 28]. The opposite directions of association of the two measures—attendance and importance—with mortality in this sample underscore the need to consider religion as a multidimensional phenomenon, and to avoid assumptions that different dimensions of religiousness will have similar associations with health [29].

This study has the limitation of relying on self-reports for health status, behaviors, and social activities. There are no biological measures, so the pathway of a direct somatic effect of religious practice or belief is unavailable to study [30, 31]. Religious attendance is known to be over-reported in social surveys [32], but such biased reporting in this case would lead to an underestimate of the effect on mortality, so it is a conservative bias. In addition, the religion measures for what are clearly complex concepts are all individual items and therefore subject to more error than would be introduced by multi-item scales that are widely available [33]. Another important limitation is the nature of the sample, which is limited to older persons. Religious attendance is lower for younger cohorts, and while self-reported “spirituality” may be higher [34], the association of this variable with mortality is not known and it is not measured in this survey.

The argument that religion should be considered among the social determinants of health in public health research and scholarship is advanced in this paper in two ways. First, the quality of the measures of wealth and net worth—the underpinnings of the social gradient—are unsurpassed in the HRS, and in a large and nationally-representative sample. The analysis shows that the hazards (protective effects) for religious attendance are certainly comparable in effect size to, or greater than, the hazards for quartiles of income and net worth.

Second, there is an interesting very large hazard for religious affiliation in the first model that is completely mediated by second and third models. In the first model, with only the religion measures, the hazard for Mainline Protestants is 293% *higher* than for respondents reporting no religious affiliation, and the other affiliations are 65–86% higher than those reporting no religion. The second model adds demographic variables, and the single addition of age to the model eliminates the differences between reporting no religion and all of the affiliations (except Conservative Protestants), because the older mean ages for all those affiliations are adjusted out. Age-adjustment reduced but did not eliminate the effect for Conservative Protestants; however, with the addition of household income and net worth in the next model the effect is fully mediated—by income and/or net worth. Conservative Protestants, thus, were older than those with no religion, but also had lower income and net worth. This suggests that the mortality hazard for Conservative Protestants is partially explained by the lower incomes and wealth of this group in a way that it is not for other religious affiliations. At the same time, the effect of attendance was minimally affected by the addition of the demographic or SES variables. Some aspects of religion, then, seem more intertwined with other social determinants, and others appear quite independent.

Our results demonstrate a prospective relationship between religion and mortality in a nationally-representative non-institutionalized adult sample that is net of, and comparable to, the association of other well-measured social determinants of health, including demographic and socioeconomic characteristics. Our study highlights the benefits of including measures of religious participation as an additional social determinant of mortality, particularly in older populations. This is a form of social engagement and identity that is of importance to many older persons in this sample and in the US population, and can provide a more complete picture of the social forces determining their health.

Data sets that have permitted the study of religion and mortality have often been limited to the single variable of attendance at services. The HRS has two additional measures of equally important dimensions of religiosity, and we see three different patterns of association: there were increased hazards related to the importance of religion, decreased hazards related to attendance, and both partially and completely mediated associations of affiliation. Our findings underscore an argument that future studies of the social determinants of health should include multiple measures of religion, tapping its complex dimensions. Religion both overlaps with, and is independent of the sources of inequality in society, and it should be considered a necessary addition to the framework.

## Supporting information

S1 Table Ordinary least squares regression of religious service attendance on other religion measures, demographics and socioeconomic status, health status, health behaviors, and social ties. Health and Retirement Study, 2004, weighted and standared errors adjusted for complex sample design.
(DOCX)

S2 Table Ordinary least squares regression of importance of religion on other religion measures, demographics and socioeconomic status, health status, health behaviors, and social ties, Health and Retirement Study, 2004, weighted and standard errors adjusted for complex sample design. (DOCX)

S3 Table Proportional hazards models for religion measures, demographics, socioeconomic status health behaviors, and social ties, with adjustment for complex sample design, Health and Retirement Study, 2004–2014. (DOCX)

S4 Table Mortality hazard ratios from Cox proportional hazard models for religion measures, separately for males and females, with successive inclusion of other social determinants, potential confounders, and potential mediators, with adjustment for complex sample design, Health. (DOCX)
