# Supplementary material for: Religion, a social determinant of mortality? A 10-year follow-up of the Health and Retirement Study
Source: PLoS One. 2017 Dec 20;12(12):e0189134. doi: 10.1371/journal.pone.0189134 (PMC5738040; doi:10.1371/journal.pone.0189134)
Supplement: S1 Table — Health and Retirement Study, 2004, weighted and standared errors adjusted for complex sample design. (DOCX) [file pone.0189134.s001.docx]

**Table S1. Ordinary least squares regression of religious service attendance on other religion measures, demographics and socioeconomic status, health status, health behaviors, and social ties, Health and Retirement Study, 2004, weighted and standard errors adjusted for complex sample design**

|  |  |  |  |  |  |
| --- | --- | --- | --- | --- | --- |
| **Variable** | **Model 1** | **Model 2** | **Model 3** | **Model 4** | **Model 5** |
| **Religion** |  |  |  |  |  |
| Religion is "very important" | .481^***^ |  |  |  |  |
| Mainline Protestant (ref.) |  |  |  |  |  |
| Conservative Protestant | .134^***^ |  |  |  |  |
| Roman Catholic | .129^***^ |  |  |  |  |
| Jewish | -.151^**^ |  |  |  |  |
| Other religion | -.171 |  |  |  |  |
| No religion | -.456^***^ |  |  |  |  |
| **Demographic characteristics** |  |  |  |  |  |
| Age in years |  | .013^***^ |  |  |  |
| Gender (female) |  | .350^***^ |  |  |  |
| Race |  |  |  |  |  |
| White (ref.) |  |  |  |  |  |
| African-American |  | .779^***^ |  |  |  |
| Other race |  | .071 |  |  |  |
| Latino |  | .521^***^ |  |  |  |
| US Born |  | -.011 |  |  |  |
| **Socioeconomic characteristics** |  |  |  |  |  |
| Education in years |  | .020^***^ |  |  |  |
| Household income (in quartiles) |  | .038^**^ |  |  |  |
| Household net assets (in quartiles) |  | .101^***^ |  |  |  |
| **Chronic conditions** |  |  |  |  |  |
| Chronic conditions (cause of death) count |  |  | -.028 |  |  |
| Chronic conditions (non-cause of death) count |  |  | .157^***^ |  |  |
| Bed days |  |  | -.006 |  |  |
| Self-rated health |  |  | .084^***^ |  |  |
| Symptoms |  |  | .017 |  |  |
| Pain |  |  | -.074^***^ |  |  |
| **Functional limitations** |  |  |  |  |  |
| ADL |  |  | -.032^**^ |  |  |
| IADL |  |  | .040^***^ |  |  |
| Sensory impairment |  |  | -.048^*^ |  |  |
| **Mental health** |  |  |  |  |  |
| CESD |  |  | -.509^***^ |  |  |
| Emotional problems |  |  | -.161^***^ |  |  |
| Memory problems |  |  | -.026 |  |  |
| **Health behaviors** |  |  |  |  |  |
| BMI |  |  |  |  |  |
| Underweight |  |  |  | -.133 |  |
| Normal weight (ref.) |  |  |  |  |  |
| Overweight |  |  |  | .021 |  |
| Obese I |  |  |  | -.005 |  |
| Obese II |  |  |  | -.041 |  |
| Smoking |  |  |  |  |  |
| Never |  |  |  | .813^***^ |  |
| Current smoker (ref.) |  |  |  |  |  |
| Former smoker |  |  |  | .476^***^ |  |
| Alcohol used days per week |  |  |  | -.094^***^ |  |
| Exercise |  |  |  | .040^***^ |  |
| Health promotion count |  |  |  | .118^***^ |  |
| **Social ties** |  |  |  |  |  |
| Marital status |  |  |  |  |  |
| Married (ref.) |  |  |  |  |  |
| Never married |  |  |  |  | -.195^*^ |
| Widowed |  |  |  |  | .139^***^ |
| Divorced/separated |  |  |  |  | -.381^***^ |
| Family size |  |  |  |  | -.001^***^ |
| Socialize frequently |  |  |  |  | .032^***^ |
| Volunteer |  |  |  |  | .542^***^ |
|  |  |  |  |  |  |
| Adjusted R^2^ | .289 | .051 | .102 | .025 | .049 |
| Constant | .786 | 1.010 | 2.485 | 1.676 | 2.439 |
| Observations, unweighted | 20,075 | 20,055 | 18,355 | 19,704 | 19,039 |

^*^ p<.05 ^**^ p<.01 ^***^ p<.001

Note: Mainline Protestant includes HRS category for Reformation Era Protestants; Conservative Protestant includes HRS categories for Pietistic, Fundamentalist, General (includes Evangelical).

Note: Cause of death chronic conditions include diabetes, cancer, lung disease, heart disease, stroke; Non-cause of death chronic conditions include hypertension, arthritis, other conditions

Note: ADL = Activities of Daily Living; IADL = Instrumental Activities of Daily Living; CESD = Centers for Epidemiologic Studies Depression scale; BMI = Body Mass Index

Note: Health promotion activities include flu shot, cholesterol test, mammogram/prostate screening, seat belt use

Note: Family size includes sum of children, grandchildren, brothers, sisters, mother, father

Note: Volunteer includes ever doing informal caregiving or volunteering for organizations
