# Supplementary material for: Religion, a social determinant of mortality? A 10-year follow-up of the Health and Retirement Study
Source: PLoS One. 2017 Dec 20;12(12):e0189134. doi: 10.1371/journal.pone.0189134 (PMC5738040; doi:10.1371/journal.pone.0189134)
Supplement: S3 Table — (DOCX) [file pone.0189134.s003.docx]

**Table S3. Proportional hazards models for religion measures, demographics, socioeconomic status, health status, health behaviors, and social ties, with adjustment for complex sample design, Health and Retirement Study, 2004-14**

|  | **Religion** | **Demographics** | **SES** | **Health status** | **Health**  **behaviors** | **Social ties** |
| --- | --- | --- | --- | --- | --- | --- |
| **Variable** | **HR (95% CI)** | **HR (95% CI)** | **HR (95% CI)** | **HR (95% CI)** | **HR (95% CI)** | **HR (95% CI)** |
| **Religion** |  |  |  |  |  |  |
| Participation |  |  |  |  |  |  |
| More than weekly | **0.44 (0.39, 0.49)** | **0.41 (0.36, 0.45)** | **0.48 (0.43, 0.54)** | **0.60 (0.53, 0.68)** | **0.74 (0.65, 0.84)** | **0.65 (0.57, 0.74)** |
| Weekly | **0.53 (0.48, 0.58)** | **0.50 (0.46, 0.55)** | **0.59 (0.53, 0.64)** | **0.73 (0.66, 0.82)** | **0.86 (0.77, 0.96)** | **0.77 (0.69, 0.86)** |
| Monthly | **0.44 (0.39, 0.50)** | **0.55 (0.49, 0.61)** | **0.62 (0.55, 0.69)** | **0.73 (0.64, 0.83)** | **0.83 (0.73, 0.95)** | **0.75 (0.66, 0.86)** |
| Yearly | **0.57 (0.52, 0.63)** | **0.71 (0.65, 0.78)** | **0.77 (0.71, 0.85)** | **0.85 (0.77, 0.95)** | 0.92 (0.83, 1.02) | **0.87 (0.79, 0.97)** |
| Never (ref.) |  |  |  |  |  |  |
| Religion is "very important" | **1.13 (1.09, 1.16)** | **1.10 (1.07, 1.13)** | **1.06 (1.04, 1.09)** | **1.04 (1.01, 1.07)** | **1.04 (1.01, 1.07)** | **1.04 (1.00, 1.07)** |
| Affiliation |  |  |  |  |  |  |
| Mainline Protestant | **3.92 (3.52, 4.36)** | 0.99 (0.89, 1.10) | 0.97 (0.87, 1.08) | 0.96 (0.85, 1.08) | 0.92 (0.81, 1.04) | 0.94 (0.83, 1.07) |
| Conservative Protestant | **1.86 (1.64, 2.12)** | **1.14 (1.02, 1.28)** | 1.06 (0.95, 1.18) | 1.03 (0.91, 1.16) | 1.02 (0.90, 1.16) | 1.05 (0.92, 1.19) |
| Roman Catholic | **1.75 (1.58, 1.95)** | 1.06 (0.97, 1.17) | 1.03 (0.96, 1.12) | 1.03 (0.92, 1.15) | 0.99 (0.88, 1.11) | 1.00 (0.89, 1.12) |
| Jewish | **1.65 (1.34, 2.03)** | 0.84 (0.69, 1.02) | 0.92 (0.75, 1.12) | 0.88 (0.70, 1.09) | 0.90 (0.73, 1.16) | 0.83 (0.67, 1.04) |
| Other religion | 1.05 (0.74, 1.50) | 0.87 (0.62, 1.23) | 0.88 (0.62, 1.23) | 0.74 (0.51, 1.08) | 0.74 (0.50, 1.10) | 0.80 (0.55, 1.17) |
| No religion (ref.) |  |  |  |  |  |  |
| **Demographic characteristics** |  |  |  |  |  |  |
| Age in years |  | **1.10 (1.09, 1.10)** | **1.09 (1.09, 1.10)** | **1.08 (1.08, 1.09)** | **1.09 (1.08, 1.09)** | **1.08 (1.08, 1.09)** |
| Gender (female) |  | **0.70 (0.66, 0.75)** | **0.63 (0.59, 0.68)** | **0.60 (0.55, 0.65)** | **0.62 (0.57, 0.68)** | **0.58 (0.54, 0.64)** |
| Race |  |  |  |  |  |  |
| White (ref.) |  |  |  |  |  |  |
| African-American |  | **1.38 (1.26, 1.52)** | 1.07 (0.97, 1.19) | 0.98 (0.87, 1.10) | 0.94 (0.84, 1.06) | 0.96 (0.86, 1.09) |
| Other race |  | 1.04 (0.85, 1.27) | 0.92 (0.75, 1.13) | 0.84 (0.67, 1.06) | 0.85 (0.67, 1.09) | 0.81 (0.64, 1.03) |
| Latino |  | 1.07 (0.92, 1.23) | **0.81 (0.69, 0.95)** | **0.84 (0.70, 0.99)** | 0.88 (0.74, 1.05) | 0.86 (0.72, 1.03) |
| US Born |  | **0.93 (0.90, 0.96)** | **0.93 (0.90, 0.96)** | **0.94 (0.90, 0.97)** | **0.94 (0.91, 0.98)** | **0.93 (0.90, 0.97)** |
| **Socioeconomic characteristics** |  |  |  |  |  |  |
| Education in years |  |  | 0.99 (0.98, 1.00) | 1.02 (1.00, 1.03) | 1.01 (1.00, 1.03) | **1.02 (1.01, 1.03)** |
| Household income (in quartiles) |  |  | **0.86 (0.83, 0.90)** | **0.92 (0.89, 0.96**) | 0.96 (0.91, 1.00) | **0.93 (0.89, 0.98)** |
| Household net assets (in quartiles) |  |  | **0.84 (0.81, 0.87)** | **0.90 (0.86, 0.93)** | **0.92 (0.88, 0.96)** | **0.92 (0.88, 0.96)** |
| **Chronic conditions** |  |  |  |  |  |  |
| Chronic conditions (cause of death) count |  |  |  | **1.21 (1.17, 1.26)** | **1.21 (1.16, 1.27)** | **1.21 (1.16, 1.26)** |
| Chronic conditions (non-cause of death) count |  |  |  | **0.93 (0.88, 0.98)** | 0.99 (0.94, 1.05) | **0.94 (0.89, 0.99)** |
| Bed days |  |  |  | **1.01 (1.00, 1.03)** | **1.01 (1.00, 1.02)** | **1.02 (1.01, 1.03)** |
| Self-rated health |  |  |  | **0.78 (0.75, 0.81)** | **0.80 (0.77, 0.84)** | **0.79 (0.76, 0.83)** |
| Symptoms |  |  |  | 1.01 (0.98, 1.04) | 1.02 (0.99, 1.05) | 1.01 (0.98, 1.04) |
| Pain |  |  |  | **0.87 (0.82, 0.91)** | **0.87 (0.82, 0.91)** | **0.87 (0.82, 0.92)** |
| **Functional limitations** |  |  |  |  |  |  |
| ADL |  |  |  | 0.97 (0.95, 1.00) | 0.97 (0.94, 1.00) | 0.97 (0.94, 1.00) |
| IADL |  |  |  | **1.12 (1.10, 1.14)** | **1.10 (1.08, 1.12)** | **1.12 (1.10, 1.14)** |
| Sensory impairment |  |  |  | **0.93 (0.88, 0.99)** | **0.93 (0.88, 0.98)** | **0.93 (0.88, 0.98)** |
| **Mental health** |  |  |  |  |  |  |
| CESD |  |  |  | 1.05 (0.88, 1.26) | 0.94 (0.79, 1.12) | 1.06 (0.89, 1.27) |
| Emotional problems |  |  |  | 0.98 (0.88, 1.08) | 1.09 (0.89, 1.36) | 0.98 (0.88, 1.09) |
| Memory problems |  |  |  | 1.05 (0.84, 1.31) | 1.16 (0.96, 1.40) | 1.03 (0.82, 1.30) |
| **Health behaviors** |  |  |  |  |  |  |
| BMI |  |  |  |  |  |  |
| Underweight |  |  |  |  | **1.47 (1.23, 1.76)** |  |
| Normal weight (ref.) |  |  |  |  |  |  |
| Overweight |  |  |  |  | **0.81 (0.75, 0.88)** |  |
| Obese I |  |  |  |  | **0.73 (0.65, 0.82)** |  |
| Obese II |  |  |  |  | 0.86 (0.74, 1.00) |  |
| Smoking |  |  |  |  |  |  |
| Never (ref.) |  |  |  |  |  |  |
| Current smoker |  |  |  |  | **1.95 (1.72, 2.21)** |  |
| Former smoker |  |  |  |  | **1.28 (1.18, 1.39)** |  |
| Alcohol used days per week |  |  |  |  | 1.01 (0.99, 1.03) |  |
| Exercise |  |  |  |  | **0.93 (0.92, 0.95)** |  |
| Health promotion count |  |  |  |  | **0.93 (0.89, 0.96)** |  |
| **Social ties** |  |  |  |  |  |  |
| Marital status |  |  |  |  |  |  |
| Married (ref.) |  |  |  |  |  |  |
| Never married |  |  |  |  |  | 1.09 (0.86, 1.38) |
| Widowed |  |  |  |  |  | 1.04 (0.94, 1.14) |
| Divorced/separated |  |  |  |  |  | 1.10 (0.96, 1.25) |
| Family size |  |  |  |  |  | 1.00 (1.00, 1.00) |
| Socialize frequently |  |  |  |  |  | 1.02 (1.00, 1.04) |
| Volunteer |  |  |  |  |  | **0.79 (0.73, 0.86)** |
|  |  |  |  |  |  |  |
| F (df), Prob > F | 123.44 (10)^***^ | 218.03 (16)^***^ | 212.78 (19)^***^ | 126.02 (310)^***^ | 99.68 (40)^***^ | 103.52 (37)^***^ |
| Observations, weighted | 78,317,290 | 78,254,220 | 78,254,220 | 71,365,979 | 69,977,879 | 67,211,961 |
| Observations, unweighted | 18,321 | 18,298 | 18,298 | 16,595 | 16,269 | 15,698 |

^*^ p<.05 ^**^ p<.01 ^***^ p<.001 **Boldface** for some estimates where the 95% CIs include 1.00 due to rounding indicates p<.05

Note: Mainline Protestant includes HRS category for Reformation Era Protestants; Conservative Protestant includes HRS categories for Pietistic, Fundamentalist, General (includes Evangelical).

Note: Cause of death chronic conditions include diabetes, cancer, lung disease, heart disease, stroke; Non-cause of death chronic conditions include hypertension, arthritis, other conditions

Note: ADL = Activities of Daily Living; IADL = Instrumental Activities of Daily Living; CESD = Centers for Epidemiologic Studies Depression scale; BMI = Body Mass Index

Note: Health promotion activities include flu shot, cholesterol test, mammogram/prostate screening, seat belt use

Note: Family size is sum of children, grandchildren, brothers, sisters, mother, father

Note: Volunteer includes ever doing informal caregiving or volunteering for organizations
