# Supplementary material for: Religion, a social determinant of mortality? A 10-year follow-up of the Health and Retirement Study
Source: PLoS One. 2017 Dec 20;12(12):e0189134. doi: 10.1371/journal.pone.0189134 (PMC5738040; doi:10.1371/journal.pone.0189134)
Supplement: S4 Table — (DOCX) [file pone.0189134.s004.docx]

**Table S4. Mortality hazard ratios from Cox proportional hazard models for religion measures, separately for males and females, with successive inclusion of other social determinants, potential confounders, and potential mediators, with adjustment for complex sample design, Health and Retirement Study, 2004-14**

|  | **Religion only** | **+Demo-graphics** | **+SES** | **+Health**  **status** | **+Health**  **behaviors** | **+Social ties** |
| --- | --- | --- | --- | --- | --- | --- |
| **Variable** | **HR**  **(95% CI)** | **HR**  **(95% CI)** | **HR**  **(95% CI)** | **HR**  **(95% CI)** | **HR**  **(95% CI)** | **HR**  **(95% CI)** |
| **FEMALES**  *Religious participation* |  |  |  |  |  |  |
| Frequently attend | **0.36**  **(0.30, 0.41)** | **0.35**  **(0.30, 0.41)** | **0.40**  **(0.34, 0.47)** | **0.54**  **(0.46, 0.65)** | **0.70**  **(0.58, 0.83)** | **0.61**  **(0.51, 0.74)** |
| Regularly attend | **0.45**  **(0.40, 0.50)** | **0.48**  **(0.43, 0.54)** | **0.54**  **(0.48, 0.61)** | **0.72**  **(0.63, 0.82)** | **0.85**  **(0.74, 0.97)** | **0.78**  **(0.68, 0.89)** |
| Occasionally attend | **0.50**  **(0.44, 0.58)** | **0.68**  **(0.59, 0.77)** | **0.72**  **(0.64, 0.82)** | **0.81**  **(0.70, 0.94)** | 0.88  (0.75, 1.02) | **0.85**  **(0.73, 0.98)** |
| Never  attend (ref.) |  |  |  |  |  |  |
| *Importance of religion* |  |  |  |  |  |  |
| Religion is "very important" | **1.19**  **(1.14, 1.24)** | **1.11**  **(1.06, 1.15)** | **1.07**  **(1.02, 1.11)** | 1.02  (0.99, 1.09) | 1.03  (0.99, 1.09) | 1.02  (0.97, 1.07) |
| *Affiliation* |  |  |  |  |  |  |
| Mainline Protestant | **4.85**  **(4.18, 5.62)** | 0.99  (0.85, 1.15) | 0.98  (0.84, 1.14) | 0.96  (0.81, 1.14) | 0.88  (0.74, 1.06) | 0.94  (0.79, 1.13) |
| Conservative Protestant | **2.12**  **(1.77, 2.54)** | **1.24**  **(1.05, 1.45)** | 1.16  (0.99, 1.36) | 1.11  (0.93, 1.32) | 1.09  (0.91, 1.31) | 1.09  (0.91, 1.31) |
| Roman Catholic | **1.77**  **(1.55, 2.02)** | 1.03  (0.91, 1.17) | 1.02  (0.89, 1.15) | 1.00  (0.86, 1.16) | 0.95  (0.81, 1.13) | 0.96  (0.83, 1.12) |
| Jewish | **1.68**  **(1.28, 2.22)** | 0.85  (0.65, 1.11) | 0.91  (0.69, 1.20) | 0.89  (0.65, 1.22) | 1.01  (0.76, 1.35) | 0.86  (0.63, 1.18) |
| Other religion | 1.27  (0.78, 2.06) | 1.08  (0.68, 1.73) | 1.05  (0.66, 1.67) | 0.84  (0.50, 1.40) | 0.80  (0.46, 1.41) | 0.88  (0.53, 1.47) |
| No religion (ref.) |  |  |  |  |  |  |
|  |  |  |  |  |  |  |
| F (df), Prob > F | 105.78 (9)^***^ | 151.44 (14)^***^ | 134.04 (17)^***^ | 75.65 (29)^***^ | 59.85 (38)^***^ | 65.37 (35)^***^ |
| Observations, weighted | 42,544,507 | 42,529,181 | 42,466,469 | 40,159,344 | 38,991,631 | 38,146,251 |
| Observations, unweighted | 10,594 | 10,588 | 10,456 | 9,922 | 9,652 | 9,326 |
|  |  |  |  |  |  |  |
|  |  |  |  |  |  |  |
|  |  |  |  |  |  |  |
|  |  |  |  |  |  |  |
| **MALES**  *Religious participation* |  |  |  |  |  |  |
| Frequently attend | **0.55**  **(0.46, 0.66)** | **0.49**  **(0.41, 0.58)** | **0.60**  **(0.50, 0.71)** | **0.69**  **(0.56, 0.84)** | **0.81**  **(0.66, 0.99)** | **0.72**  **(0.59, 0.88)** |
| Regularly attend | **0.56**  **(0.449, 0.64)** | **0.55**  **(0.49, 0.63)** | **0.67**  **(0.59, 0.76)** | **0.75**  **(0.65, 0.87)** | **0.85**  **(0.73, 0.99)** | **0.76**  **(0.65, 0.89)** |
| Occasionally attend | **0.65**  **(0.56, 0.74)** | **0.75**  **(0.66, 0.86)** | **0.83**  **(0.73, 0.95)** | 0.90  (0.78, 1.04) | 0.97  (0.84, 1.12) | 0.90  (0.78, 1.04) |
| Never  attend (ref.) |  |  |  |  |  |  |
| *Importance of religion* |  |  |  |  |  |  |
| Religion is "very important" | **1.12**  **(1.08, 1.16)** | **1.09**  **(1.05, 1.14)** | **1.06**  **(1.02, 1.10)** | **1.04**  **(1.00, 1.09)** | 1.04  (1.00, 1.08) | **1.05**  **(1.00, 1.09)** |
| *Affiliation* |  |  |  |  |  |  |
| Mainline Protestant | **3.45**  **(2.94, 4.06)** | 0.99  (0.85, 1.16) | 0.97  (0.83, 1.13) | 0.96  (0.81, 1.14) | 0.95  (0.80, 1.12) | 0.95  (0.80, 1.15) |
| Conservative Protestant | **1.73**  **(1.44, 2.09)** | 1.07  (0.91, 1.26) | 0.96  (0.82, 1.13) | 0.97  (0.82, 1.15) | 0.97  (0.82, 1.15) | 1.02  (0.86, 1.22) |
| Roman Catholic | **1.82**  **(1.54, 2.14)** | 1.09  (0.94, 1.26) | 1.02  (0.88, 1.18) | 1.04  (0.89, 1.22) | 1.01  (0.87, 1.19) | 1.04  (0.89, 1.22) |
| Jewish | **1.68**  **(1.23, 2.31)** | 0.82  (0.61, 1.09) | 0.91  (0.68, 1.22) | 0.83  (0.61, 1.14) | 0.79  (0.57, 1.10) | 0.79  (0.57, 1.10) |
| Other religion | 0.89  (0.53, 1.50) | 0.70  (0.43, 1.14) | 0.73  (0.44, 1.19) | 0.62  (0.36, 1.06) | 0.66  (0.39, 1.11) | 0.69  (0.40, 1.21) |
| No religion (ref.) |  |  |  |  |  |  |
|  |  |  |  |  |  |  |
| F (df),  Prob > F | 43.66 (9)^***^ | 98.19 (14)^***^ | 105.61 (17)^***^ | 63.20 (29)^***^ | 51.63 (38)^***^ | 50.49 (35)^***^ |
| Observations, weighted | 35,772,783 | 35,725,039 | 35,641,304 | 31,206,635 | 30,986,248 | 29,065,710 |
| Observations, unweighted | 7,848 | 7,831 | 7,818 | 6,794 | 6,738 | 6372 |

^*^ p<.05 ^**^ p<.01 ^***^ p<.001 **Boldface** indicates p<.05, including for some estimates where the 95% CIs apparently include 1.00 due to rounding

Note: Mainline Protestant includes HRS category for Reformation Era Protestants; Conservative Protestant includes HRS categories for Pietistic, Fundamentalist, General (includes Evangelical).

Note: Cause of death chronic conditions include diabetes, cancer, lung disease, heart disease, stroke; Non-cause of death chronic conditions include hypertension, arthritis, other conditions

Note: ADL = Activities of Daily Living; IADL = Instrumental Activities of Daily Living; CESD = Centers for Epidemiologic Studies Depression scale; BMI = Body Mass Index

Note: Health promotion activities include flu shot, cholesterol test, mammogram/prostate screening, seat belt use

Note: Family size is sum of children, grandchildren, brothers, sisters, mother, father

Note: Volunteer includes ever doing informal caregiving or volunteering for organizations
